# Supplementary material for: Recent acceleration in global ocean heat accumulation by mode and intermediate waters
Source: Nat Commun. 2023 Oct 28;14:6888. doi: 10.1038/s41467-023-42468-z (PMC10613216; doi:10.1038/s41467-023-42468-z)
Supplement: Supplementary file 1 — Supplementary Information [file 41467_2023_42468_MOESM1_ESM.pdf]

Supplementary Information for

Recent acceleration in global ocean heat accumulation by mode and intermediate waters

Zhi Li[1,2,3*], Matthew H. England[2,3], and Sjoerd Groeskamp[4]

[1]Climate Change Research Centre, University of New South Wales, NSW 2052, Australia.

[2]Australian Centre for Excellence in Antarctic Science, University of New South Wales, NSW 2052, Australia.

[3]Centre for Marine Science and Innovation (CMSI), University of New South Wales, NSW 2052, Australia.

[4]NIOZ Royal Netherlands Institute for Sea Research, Department of Ocean Systems, 1790 AB, Den Burg, Texel, The Netherlands.

[*]Corresponding Author, e-mail: zhi.li4@unsw.edu.au.

Contents:

- Supplementary Figures 1–3
- Supplementary Tables 1–4

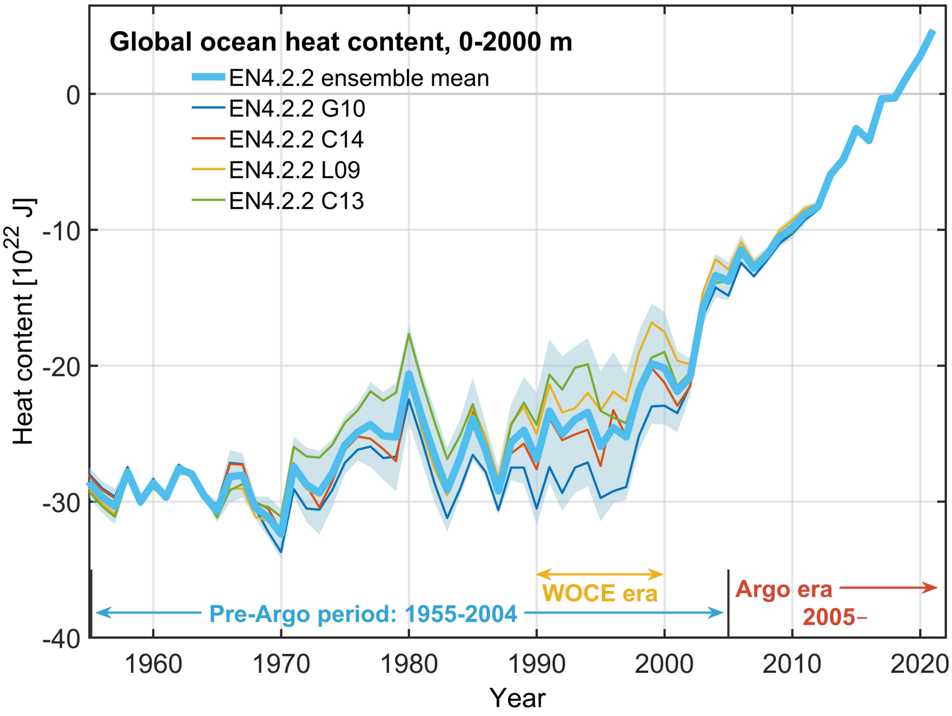

Supplementary Figure 1. Time series for the heat content of the upper 2000 m of the ocean relative to 2016–2020 mean, using EN4.2.2 data (ref. [32]). Units are shown as 10^{22} J. Thin lines indicate the ocean heat content (OHC) anomaly from four ensemble members of EN4.2.2 data, bold blue line represents the ensemble mean time series of global OHC during 1955–2020, and shading indicates the ± 2 ensemble standard deviation uncertainty range ($\pm 2\sigma$).

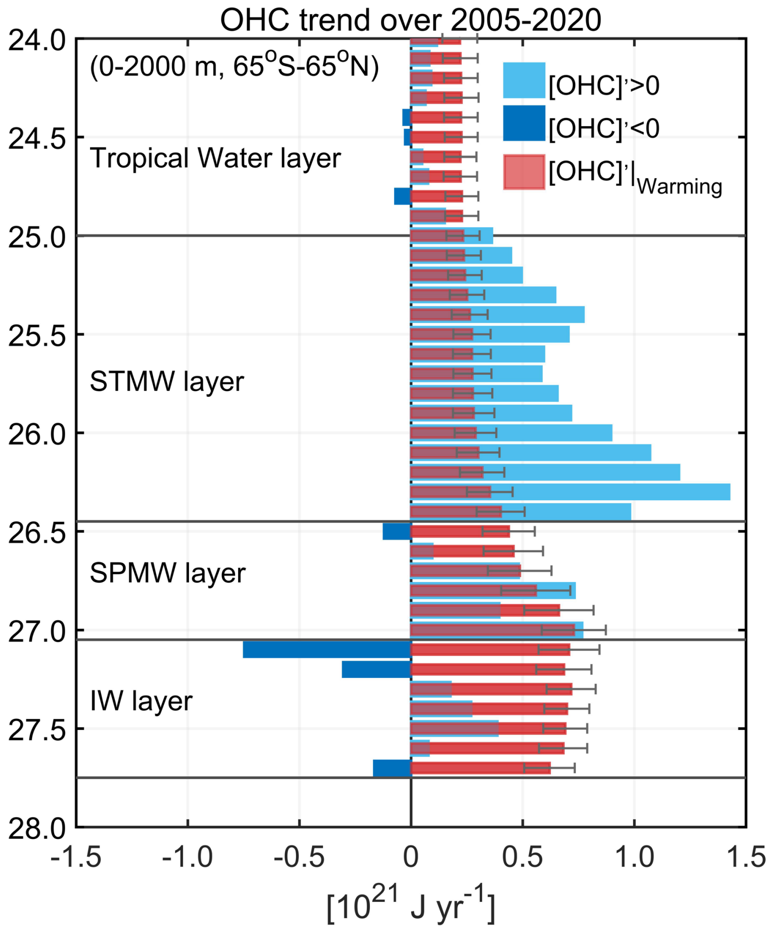

Supplementary Figure 2. Global ocean heat content (OHC) change distributed by density layers. The increase (light blue) and decrease (dark blue) of OHC are shown for every $\gamma^{\mathrm{n}} = 0.1\ \mathrm{kg\,m^{-3}}$ isopycnal layers between 24.0 and $27.7\ \mathrm{kg\,m^{-3}}$ ($10^{21}\ \mathrm{J\ yr^{-1}}$ per $0.1\ \mathrm{kg\,m^{-3}}$ density bin), 2005–2020. Red bars indicate the warming component of OHC change (Methods). Bars represent the ensemble average of OHC trends from SIO RG-Argo, IAP data, and EN4.2.2 ensemble mean, and superimposed error bars indicate the ± 1 ensemble standard deviation uncertainty range ($\pm 1\sigma$).

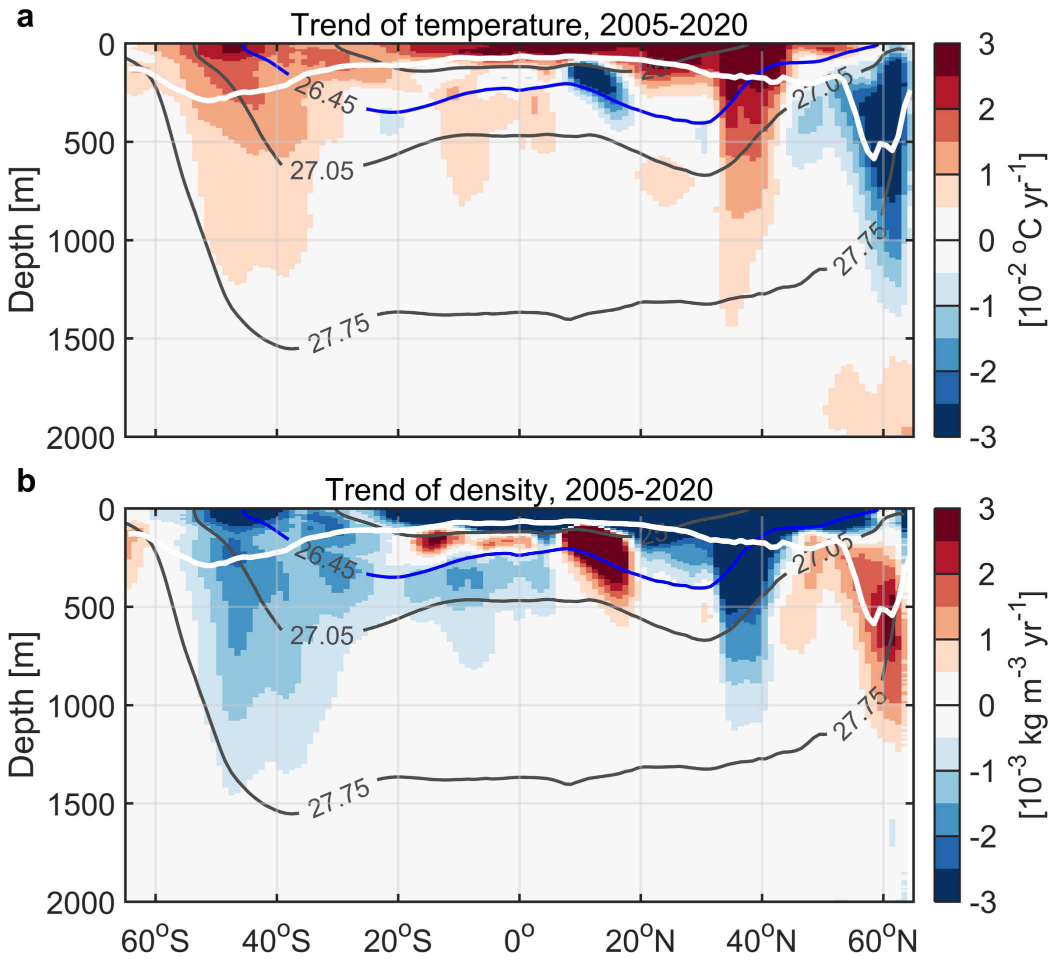

Supplementary Figure 3. Linear trends in zonally averaged temperature and density over the Argo era. Trends in zonally averaged (**a**) Conservative Temperature (10^{-2} °C yr^{-1}) and (**b**) neutral density (10^{-3} Kg m^{-3} yr^{-1}). The results presented in panels (**a-b**) represent the ensemble means from SIO RG-Argo, IAP data, and EN4.2.2 ensemble mean. Superimposed dark-grey contours represent the wintertime isopycnals of $\gamma^n = 25$, 26.45, 27.05 and 27.75 kg m^{-3} from SIO RG-Argo, white line represents the zonally averaged wintertime mixed-layer depth over the Argo era.

Supplementary Table 1 | OHC trend in water-mass layers, 2005–2020		
	OHC trend ($\times 10^{21}$ J yr^{-1})	Density range (γ^n, kg m^{-3})
0–2000 m	9.7±1.8	All
Tropical water layer	1.0±1.5	γ^n <25.0
Mode water layer	10.0±1.9	25.0≤ γ^n <27.05
Intermediate water layer	−1.5±1.5	27.05≤ γ^n <27.75
Deep water and bottom water layers	0.8±1.6	27.75≤ γ^n

Supplementary Table 1 | Ocean heat content (OHC) trend of the upper 2000 m separated by water-mass layers over 2005–2020. Units are shown as 10^{21} J yr^{-1}. Heat content trends of the upper 2000 m of the ocean, tropical water layer, mode water layer, intermediate water layer, and deep and bottom water layers. The results presented indicate the ensemble mean and $\pm 2\sigma$ from SIO RG-Argo, IAP data, and EN4.2.2 ensemble mean. Note that the global OHC trend of the upper 2000 m over 2005–2020 in **Table 1** additionally includes the OHC trend estimate from ref. [8,31], and the estimate of OHC trends in **Supplementary Table 1** is limited to 65°S-65°N.

Supplementary Table 2 | Warming and thickening of water-mass layers			
Water-mass layer	OHC trend ($\times 10^{21}$ J yr^{-1})	Volume, $\partial\rho/\partial t$ ($\times 10^{21}$ J yr^{-1})	Temperature, $\partial T/\partial t$ ($\times 10^{21}$ J yr^{-1})
0–2000 m	9.7±1.8	0.0±0.0	9.7±1.8
Tropical water layer	1.0±1.5	−0.5±1.3	1.4±0.2
Mode water layer	10.0±1.9	6.3±0.9	3.8±1.1
Intermediate water layer	−1.5±1.5	−5.1±1.3	3.7±0.7
Mode and intermediate water layers	8.6±2.2	1.2±0.8	7.4±1.7
Deep water and bottom water layers	0.8±1.6	−0.7±1.4	1.5±0.6

Supplementary Table 2. Heat content trend in global mode and intermediate water layers due to warming and volumetric change components, 2005–2020. Units are shown as 10^{21} J yr^{-1}. Same as **Supplementary Table 4** but for global mode and intermediate water layers as sketched in **Fig. 4e** (**Supplementary Table 1**; Methods). The ensemble averaged ocean heat content (OHC) trends and the associated ± 2 ensemble standard deviation uncertainty range ($\pm 2\sigma$) were obtained from SIO RG-Argo, IAP data, and EN4.2.2 ensemble mean, and were limited to 65°S-65°N.

Supplementary Table 3 | Heat content trend in mode and intermediate waters				
**Heat content change**		**Constraints for defining mode and intermediate waters**		
Water mass	OHC trend ($\times 10^{21}$ J yr^{-1})	Density range (γ^n, kg m^{-3})	Longitude range	Latitude range
0–2000 m	9.7±1.8	All	65°S - 65°N	0° - 360°
SAMW	3.8±0.4	26.45≤ γ^n<27.05	65°S - 30°S	0° - 360°
SA-AAIW	0.7±0.4	27.05≤ γ^n<27.75	65°S - 0°	60°W - 20°E
SP- and SI-AAIW	−2.2±0.5	27.05≤ γ^n<27.75	65°S - 30°S	20°E - 60°W
SA-STMW	0.4±0.2	25.6≤ γ^n<26.45	50°S - 20°S	70°W - 20°E
NA-STMW	3.6±0.7	25.0≤ γ^n<26.45	15°N - 45°N	80°W - 30°W
NA-MMW	−2.6±0.5	26.45≤ γ^n<26.95	15°N - 45°N	80°W - 30°W
NA-SPMW	−1.0±0.1	26.95≤ γ^n<27.75	40°N - 65°N	40°W - 0°
SP-WSTMW	1.1±0.2	25.25≤ γ^n<26.45	42°S - 20°S	150°E - 150°W
SP-ESTMW	0.7±0.3	25.0≤ γ^n<25.7	30°S - 10°S	150°W - 80°W
NP-STMW	0.2±0.4	25.0≤ γ^n<25.7	20°N - 35°N	130°E - 160°W
NP-ESTMW	−0.5±0.2	24.0≤ γ^n<25.5	20°N - 40°N	160°W - 120°W
NP-CMW	0.2±0.1	25.7≤ γ^n<26.5	30°N - 45°N	140°E - 150°W
NP-IW	−0.1±0.1	26.5≤ γ^n<27.4	20°N - 65°N	130°E - 135°W
IO-STMW	−0.04±0.03	25.8≤ γ^n<26.3	40°S - 27°S	25°E - 60°E
All Water Masses	4.3±1.4	-	-	-

Supplementary Table 3. Heat content change in regionally defined mode and intermediate waters. Units are shown as 10^{21} J yr^{-1}. The first two columns correspond to bars in **Fig. 8e**; the specific density and geographic constraints for defining individual mode and intermediate waters are listed in columns 3–5. The ensemble mean estimate of ocean heat content (OHC) trends and the associated ± 2 ensemble standard deviation uncertainty range ($\pm 2\sigma$) in column 2 were obtained from SIO RG-Argo, IAP data, and EN4.2.2 ensemble mean.

Supplementary Table 4 | Warming and thickening of water masses			
Water mass	OHC trend ($\times 10^{21}$ J yr^{-1})	Volume, $\partial\rho/\partial t$ ($\times 10^{21}$ J yr^{-1})	Temperature, $\partial T/\partial t$ ($\times 10^{21}$ J yr^{-1})
0–2000 m	9.7±1.8	0.0±0.0	9.7±1.8
SAMW	3.8±0.4	2.4±0.2	1.4±0.4
AAIW	−1.4±0.4	−3.6±0.8	2.1±0.2
SA-STMW	0.4±0.2	0.2±0.2	0.1±0.0
NA-STMW	3.6±0.7	3.2±0.6	0.4±0.1
NA-MMW	−2.6±0.5	−2.9±0.6	0.3±0.1
NA-SPMW	−1.0±0.1	−0.4±0.0	−0.7±0.1
SP-WSTMW	1.1±0.2	0.8±0.2	0.3±0.1
SP-ESTMW	0.7±0.3	0.6±0.3	0.1±0.0
NP-STMW	0.2±0.4	0.2±0.4	0.0±0.0
NP-ESTMW	−0.5±0.2	−0.6±0.2	0.1±0.0
NP-CMW	0.2±0.1	0.0±0.2	0.2±0.1
NP-IW	−0.1±0.1	−0.4±0.2	0.3±0.1
All Water Masses	4.3±1.4	−0.4±0.8	4.7±0.9

Supplementary Table 4. Heat content change in regionally defined mode and intermediate waters due to warming and volumetric change components, 2005–2020. Units are shown as 10^{21} J yr^{-1}. The last three columns correspond to bars in **Fig. 9g**, indicating the total ocean heat content (OHC) trend and its components by volumetric change ($\partial\rho/\partial t$) and temperature change ($\partial T/\partial t$). The ensemble mean estimate of OHC trends and the associated ± 2 ensemble standard deviation uncertainty range ($\pm 2\sigma$) were obtained from SIO RG-Argo, IAP data, and EN4.2.2 ensemble mean.
